# Modeling AIDS survival after initiation of antiretroviral treatment by Weibull models with changepoints

**DOI:** 10.1186/1758-2652-12-9

**Published:** 2009-06-26

**Authors:** Constantin T Yiannoutsos

**Affiliations:** 1 Division of Biostatistics, Indiana University School of Medicine,401 W. 10th Street, Suite 3000, Indianapolis, USA

## Abstract

**Background:**

Mortality of HIV-infected patients initiating antiretroviral therapy in the developing world is very high immediately after the start of ART therapy and drops sharply thereafter. It is necessary to use models of survival time that reflect this change.

**Methods:**

In this endeavor, parametric models with changepoints such as Weibull models can be useful in order to explicitly model the underlying failure process, even in the case where abrupt changes in the mortality rate are present. Estimation of the temporal location of possible mortality changepoints has important implications on the effective management of these patients. We briefly describe these models and apply them to the case of estimating survival among HIV-infected patients who are initiating antiretroviral therapy in a care and treatment programme in sub-Saharan Africa.

**Results:**

As a first reported data-driven estimate of the existence and location of early mortality changepoints after antiretroviral therapy initiation, we show that there is an early change in risk of death at three months, followed by an intermediate risk period lasting up to 10 months after therapy.

**Conclusion:**

By explicitly modelling the underlying abrupt changes in mortality risk after initiation of antiretroviral therapy we are able to estimate their number and location in a rigorous, data-driven manner. The existence of a high early risk of death after initiation of antiretroviral therapy and the determination of its duration has direct implications for the optimal management of patients initiating therapy in this setting.

## Background

In a clinical trial, 1120 HIV-infected individuals initiated antiretroviral therapy (ART) in rural Uganda. One hundred and five subjects died during the study. Table 1 shows the number of patients dying per 100 person-years of follow up in every period post ART initiation. Inspection of the mortality rates in Table 1 suggests that the risk of mortality (hazard) immediately after ART initiation is higher than the hazard in later periods. The possibility that there is a point in time that the hazard of death changes abruptly, from an early high level to a lower level, has broad implications for the management of these patients. A number of reports have indicated that mortality risk is higher immediately following the start of therapy [1-3]. These reports consider that the period of high mortality lasts about three to six months after start of therapy. To analyze this type of survival data, acknowledgement of the possibility of a sharp decline in the hazard of death is essential both for the analysis itself, and in a data-driven manner, for the probable location of the changepoint of mortality risk. In the problem discussed here, it is useful to derive objective data-driven estimates of the number and temporal location of these risk changepoints, since their existence has broad implications on clinical protocols developed for the management of these patients. The class of Weibull models with changepoints is suitable for this purpose as these models explicitly model the underlying hazard of mortality and, therefore, are useful in better understanding the disease process.

**Table 1:** Death rate (per 100 person-years) after initiation of ART

	Time period				
	
	< 3 months	3–6 months	6–12 months	12–18 months	18–24 months
Subjects at interval start	1,120	1,074	1,036	994	959
Deaths	45	15	23	13	6
Patients lost	1	23	19	22	20
Person years	273.8	266.2	508.7	491.6	474.7
Death rate	16.4	5.6	4.5	2.7	1.3

## Methods

### The Weibull model of patient survival

A frequently used mathematical model of patient survival is based on the Weibull probability distribution function for survival time *T*. The baseline hazard of this model for the *i*^th ^subject is

Covariates are incorporated straightforwardly so that *h*(*t*_*i*_; **z**) = *h*_0_(*t*_*i*_)*e*^*β'***z**^, i.e.,

where *β *and **z **are the vectors of coefficients and covariate values respectively. The cumulative hazard of the Weibull model from the time origin up to time *t*_*i *_is

so that

This is a proportional hazards model in the sense that the hazard at time *t*_*i*_, given the covariates **z**, is *h*(*t*; **z**) = *h*_*o*_(*t*)*ϕ *where *ϕ *= *e*^*β'***z **^is a proportionality constant that is not dependent on time. The log cumulative hazard is also linear in both time and the covariates, i.e., log *H*(*t*_*i*_; **z**) = *ρ*log *t*_*i *_- *ρ*log *λ *+ *β'***z **or, in the notation of Royston and Parmar [4],(1)

where *s*(*x*; *γ*) = *γ*_0 _+ *γ*_1_*x *with *γ*_0 _= -*ρ*log *λ*, *γ*_1 _= *ρ *and *x *= log *t*_*i*_. The Weibull model is a generalization of the common exponential survival model (having *ρ *= 1). It is more flexible for many real-world situations as, in contrast to the exponential model, it does not assume constant hazard of death.

### Extensions of the Weibull model

As flexible as the Weibull model may be, it may not adequately reflect changes in the hazard over time such as in the case of very high mortality hazard early after the start of ART. We consider one such extension, which includes Weibull models with changepoints.

#### Weibull model with changepoints

The general *m*-changepoint model for the Weibull is given by Noura and Read [5]. They consider the baseline log cumulative hazard for the *i*^th ^subject

where

are changepoint indicators. Ensuring that the piece-wise log-cumulative hazards (and thus the piece-wise survival curves) meet at the changepoints *a*_*j*_, for *j *= 0,⋯,*m *+ 1 (with *a*_0 _= 0 and *a*_*m*+1 _= ∞) puts restrictions on the *λ*_*j *_parameters of the *m *piece-wise Weibull distributions making up the model. Details can be found in the Noura and Reed [5] analysis. The usual logarithm of the survival likelihood (i.e., the probability that the death and censoring times will be as observed in the data), obtained from the piece-wise Weibull model is(2)

where *δ*_*i *_is a censoring indicator and(3)

In the simplest case of a single changepoint *a*, we will have two Weibull models, before and after the changepoint, with scale and shape parameters (*λ*_1_, *ρ*_1_) and (*λ*_2_, *ρ*_2_) respectively. For a given single changepoint *a*, the log likelihood is, by (2)(4)

The log cumulative hazard log *H*(*t*_*i*_) is obtained by adapting equation (3) for the case of a single changepoint. Doing so we obtain the log cumulative hazard for the single changepoint model

where the changepoint indicators in (3) are, in the case of two changepoints, *c*_1*i *_= *c*_*i *_and *c*_2*i *_= (1 - *c*_*i*_). The likelihood in (4) is called the log *profile *likelihood associated with a particular choice of changepoint *a*. The analysis proceeds by maximizing (4), with respect to *ρ*_1_, *ρ*_2 _and *λ*_1_, for given candidate values of the changepoint *a*. Repeating this process over a number of candidate changepoints and maximizing the log profile likelihood for each one, we can determine the optimal changepoint *a *and the maximum profile likelihood estimates of the piece-wise Weibull distributions  and  with  log *a*. For more details on the single-changepoint Weibull model, see [6].

In the case of the Weibull model with two changepoints *a*_1 _and *a*_2_, the log profile likelihood is(5)

where

is the log cumulative hazard, and *δ*_*i*_, *c*_*i*1_, *c*_*i*2 _and *c*_*i*3 _= 1 - *c*_*i*1 _- *c*_*i*2 _are, respectively, the censoring and changepoint indicators. This profile likelihood can be maximized with respect to *ρ*_1_, *ρ*_2_, *ρ*_3 _and *λ*_1_, over a grid of candidate changepoint values *a*_1 _and *a*_2 _until the maximum profile likelihood is found. This results in the determination of the optimal changepoints *a*_1 _and *a*_2 _as well as the maximum profile likelihood estimates of the three piece-wise Weibull distributions, ,  and  where  log *a*_1 _and  log *a*_2 _[5].

#### Inference on the changepoints

The location of the changepoint produced by the methods just outlined does not account for the variability associated with the estimation procedure. A way to produce confidence intervals (in the case of a single changepoint) or confidence regions (in multiple dimensions) is by inverting the likelihood ratio test [7,8]. That is, the confidence region is comprised of all changepoints **a **satisfying

where *m *is the number of changepoints,  is the 1 - *α *percentile of the chi-square distribution with *m *degrees of freedom, *L*(**a**) is the maximized log-likelihood function evaluated at changepoint **a **and *L*(**â**) is the maximized log-likelihood function evaluated at the optimal changepoint. The authors of the aforementioned references contend that these confidence intervals will perform well even if the underlying likelihood is not normal.

#### Incorporating covariates

As shown earlier, factors that are thought to be associated with the mortality hazard are straightforwardly incorporated into the model. Estimation of the regression coefficient *β*, associated with one or more factors, proceeds through maximizing the likelihood equation in (4) or (5), depending on the model selected. *β *is an additional parameter for maximization. The estimate of *β *that maximizes the profile likelihood, at the optimal value of the changepoint is the maximum likelihood estimator  (see [8] for example). As usual, the hazard ratio is equal to *e*^*β*^. Variance estimates for , produced by the inversion of the information matrix associated with the profile likelihood, are generally not adequate, since they do not reflect the uncertainty introduced by estimating the changepoints. The conditional variance formula can be used in this case [8], i.e.,(6)

The first term on the right of equation (6) is the average of the variance of  and the second is the variance of the average estimate of . Both the estimate of each  and its variance are readily produced in the output of most statistical software packages used to implement the analysis

#### Model comparison

Selecting the optimum model among those with the same number of changepoints can be accomplished, by performing a grid search and evaluating the profile likelihood at a number of candidate changepoints and selecting the one that maximizes the likelihood.

Selecting the optimal model among models with different numbers of changepoints can be accomplished by comparing the Akaike or Bayesian Information Criterion (abbreviated as AIC and BIC respectively). Both of these include a penalty against over parametrization of the model. Thus, in both cases, the model with the lowest AIC or BIC is preferred. The AIC criterion is given by the relationship *AIC *= 2*m *- 2 log(*L*), where *m *is the dimension of the model and -2 log(*L*) is minus 2 times the logarithm of the maximized likelihood at model convergence (also called the *deviance *because it is a measure of the deviation of the model from the data). The AIC is distributed according to a chi-square distribution for large samples. The BIC criterion is similar to the AIC in that it penalizes models with larger number of changepoints. The BIC is given by the general equation *BIC *= *m *log(*n*) - 2 log(*L*), where *n *is the number of observations in the model. This may not be correct in the special case of survival analysis where subjects provide varying amounts of data depending on whether they have been observed to die or they are censored as of the end of the study. We will follow the recommendation of Volinsky and Raftery [9] and substitute *d*, the number of deaths, for *n*, in the equation for the BIC. It should be noted that, by the definition of the AIC and the BIC, among models with the same dimension, the one maximizing the likelihood is the optimum model with respect to both the AIC and BIC.

All analyses involving Weibull models with changepoints were performed using the ml command in Stata version 9.2 (Stata Corporation, College Station, TX). The author will make the programme code available upon request.

## Results

### The Home-based AIDS Care Programme

The Home-Based AIDS Care Programme (HBAC) [10] is a clinical trial examining three different monitoring strategies for HIV-infected patients receiving ART in rural Uganda. Aggregated data with no information on any of the three monitoring strategies were used for this analysis. The HBAC study was approved by the Science and Ethics Committee of the Uganda Virus Research Institute, the Institutional Review Boards of the Centers for Disease Control and Prevention and the University of California, San Francisco. In total, 1120 HIV-infected patients were administered antiretroviral medications as part of the study. The duration of follow-up in this patient cohort was as short as 10 days and as long as almost 33 months (median 26.9 months, inter-quartile range 23.9–29.9 months). One hundred and five subjects (cumulative mortality 9.38%) died in the study after initiation of ART. Mortality rates over various periods of the study are summarized in Table 1. Over the first two years of follow-up, 95 patients discontinued from the study (cumulative two-year dropout probability 7.6%). Because of the very low number of patients who were lost to follow-up during the study, these data are particularly useful as an illustration of these methods because they are not burdened by possible biases resulting from differential vital status assessment of the subjects in the research cohort. This is a serious problem with cohorts in the same context [11].

### Weibull analysis of the Uganda mortality data

**Figure 1:**
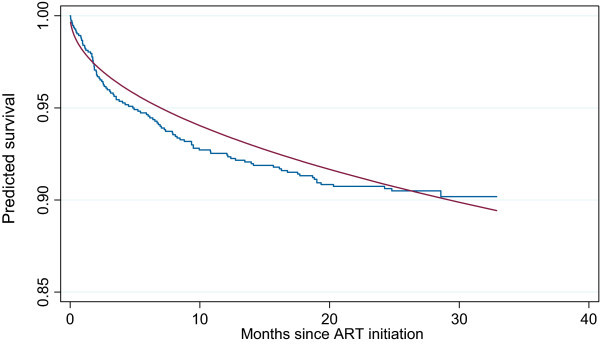
**Kaplan Meier (step function) versus Weibull estimates of patient survival (smooth curve)**. Two alternative analyses of the HBAC survival data

A Weibull analysis of the study data is compared to the Kaplan-Meier estimate of patient survival in Figure 1. It is clear from the figure, that the Weibull model underestimates patient mortality immediately following ART initiation. For this reason it would be useful to consider more flexible models that take into consideration possible changes in hazard over various periods after initiation of therapy. In addition, detection of times where the risk of death changes sharply (changepoints), has broad implications for the management of these patients.

### Reanalysis of the data by Weibull models with changepoints

The data can be re-analyzed by using more flexible Weibull models with one or more changepoints.

#### Weibull analysis with a single changepoint

To carry out this analysis, we maximize the log profile likelihood shown in equation (4) for a number of candidate early changepoints *a*. We considered, as candidate points, any month within the first year after initiation of ART. This was a deliberate choice since a single changepoint after the first year would be of limited utility for care purveyors.

**Figure 2:**
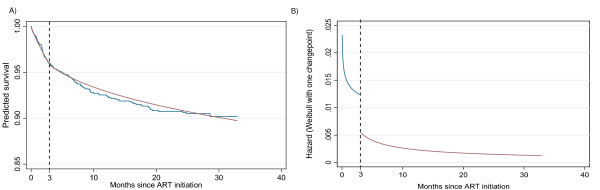
**Survival estimates produced by Kaplan Meier versus a Weibull model with one changepoint (left panel) and hazard plot of the Weibull single-changepoint model (right panel)**. These are the estimates of the survival produced by the Weibull model with one changepoint (left panel). This panel is like the one on Figure 1 but with a "kink" in the Weibull curve. The hazard plot of the Weibull single-changepoint model is also given (right panel).

The log profile likelihood has a maximum at *a *= 3. This means that the model with a changepoint in survival three months after initiation of antiretroviral therapy (95% confidence interval 2.1–4.3 months), is the best single-changepoint model. The estimated Weibull survival is shown in Figure 2 along with the Kaplan-Meier reference survival estimate (left panel).

The impression is that the fit, particularly in the period after the first three months, is particularly good, but the survival estimate still underestimates the mortality rate in the later period after initiation of ART. Another informative figure of the implication of the changepoint model is the hazard plot shown in the right panel of the Figure 2. This single-changepoint model reflects a situation of a very high hazard of death in the first three months after ART initiation, followed by a period of lower hazard. It is also worth noting that the construction of the model ensures that the individual cumulative hazard curves, and thus the survival curves before and after the changepoint, will meet resulting in a continuous survival curve. This, however, is not the case with the hazard curves that are discontinuous at the changepoint as a byproduct of the model construction.

#### Weibull analysis with two changepoints

To address the poor fit in the middle part of the follow-up period, we add one more changepoint to the Weibull model. To fit the two-changepoint model, we must maximize the profile likelihood from equation (5) presented in the Methods section, for given candidate changepoints *a*_1 _and *a*_2 _searching through various combinations of candidate changepoints. We considered, as candidate points, any month within the 18 months after initiation of ART for both the first and second changepoint.

Performing this analysis, the optimal two changepoints were found to be at *a*_1 _= 3 and *a*_2 _= 10 months after initiation of ART.

**Figure 3:**
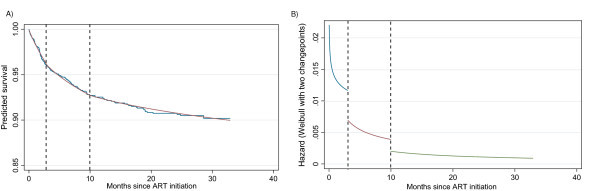
**Survival estimates produced by Kaplan Meier versus a Weibull model with two changepoints (left panel) and hazard plot of the Weibull two-changepoint model (right panel)**. This figure is similar to the one presented in Figure 2 only the Weibull model with two changepoints (left panel) is now presented. The hazard plot of the Weibull two-changepoint model is also shown (right panel).

**Figure 4:**
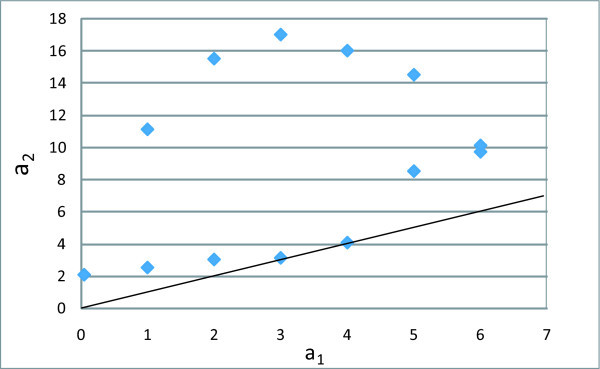
**Confidence region based on the inversion of the likelihood ratio test for the Weibull two-changepoint model. The straight line is the 45-degree diagonal. Any points below the diagonal would imply that a second changepoint is located earlier than the first changepoint. No such points were within the confidence region**. This figure shows the 95% confidence region for the two-changepoint model.

The new survival estimate is shown in Figure 3 (left panel). The fit from the two-changepoint Weibull model is very good throughout the post-ART period. A hazard plot of the two-changepoint problem is shown in the right panel of Figure 3. The hazard plot implies that there are three periods after initiation of ART. The first is the initial period of high risk immediately after initiation of ART that extends up to three months from start of therapy, followed by the second, an intermediate risk period between three and 10 months. This is itself followed by a period of stabilized (almost constant) low risk of death, starting 10 months after therapy initiation. A 95% confidence region is given in Figure 4, and is produced by varying vector **a **= (*a*_1_, *a*_2_) around **â **= (3, 10) and considering the region where . The interpretation is less straightforward than in the one-dimensional case. Considering the two optimal values of the first and second changepoints, the 95% confidence interval for the first changepoint at *a*_2 _= 10 is between approximately one and six months. The 95% confidence interval for the second changepoint at *a*_1 _= 3 is approximately between three and 16.5 months. While not guaranteed by the construction of the model, the confidence region, reassuringly, does not include any points that would support a second changepoint that is temporally earlier than the first, i.e., there are no points below the 45-degree diagonal.

#### Model comparison

Comparing the two best Weibull models with a one and two changepoints via the Akaike Information Criterion (AIC) and the Bayesian Information Criterion (BIC) (see Methods section) produces AIC values of 1296.7 and 1293.3 for the one and two-changepoint models respectively and BIC values of 1304.6 and 1303.9 respectively. This means that the model with the two changepoints is superior according to both the AIC and BIC.

#### Incorporating covariates

As an illustration of incorporating covariates into the Weibull models with changepoints, we present the analysis of the post-ART survival of male versus female patients. There were 815 women in the data set (72.8% of the study cohort) compared to 305 men. Maximizing the log profile likelihood in (5), with gender as the covariate, adds *β*, the associated survival regression coefficient, as an additional parameter for maximization. Performing this analysis, the optimal changepoint model is the one with two changepoints at *a*_1 _= 3 and *a*_2 _= 10 months post ART start. The estimate of the Weibull regression coefficient is  = 0.411, which corresponds to a hazard ratio of 1.51 of male compared to female patients.

Following the conditional variance estimation approach in (6), we obtain var() = 0.0417. This in turn implies that a 95% confidence interval of the hazard ratio will be (1.01, 2.25). The Wald p value is *p *= 0.041 indicating an increase in the hazard of mortality among men compared to women. As it turned out in this application, . However, this will not be the case universally. 

**Figure 5:**
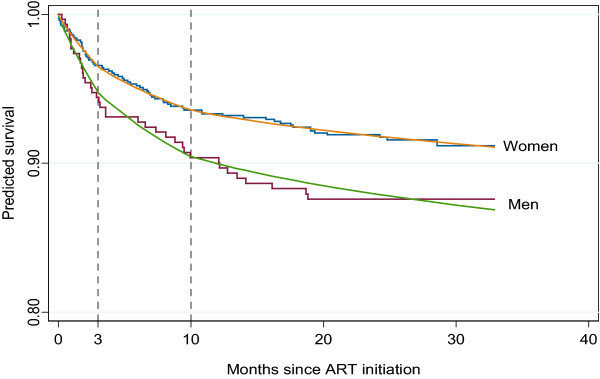
**Survival estimates produced by Kaplan Meier versus a Weibull model with two changepoints for male and female patients**. The figure is similar to the one presented in the left panel of Figure 3 but includes a stratification by gender to illustrate the methodology when subject subgroups are considered.

The results from this analysis are shown pictorially in Figure 5. We should note that the model forced the changepoints to be at three and 10 months for both groups. We also considered alternative analyses where the data for men and women were fit separately and the optimal changepoints were determined. There was no evidence to suggest that the changepoints for men and women were different.

## Discussion

The main goal of this research is to establish, in a data-driven manner, the existence, temporal location and number of sharp changes in mortality risk (hazard of death) after initiation of ART in a care and treatment programme in sub-Saharan Africa. A number of investigations have reported that a high risk of death persists for some time after ART initiation compared to later periods [1-3]. Establishing the duration of this high risk period is significant for refining clinical care protocols to better manage these patients. For example, the frequency of patient visits can be intensified for high-risk individuals and patient counselling and outreach can also be considered over this crucial period.

The existence of a changepoint of risk has been empirically placed at some time during the first three to six months of therapy by a number of reports. To my knowledge however, there has never been an objective estimate of its location generated by rigorous data analysis. In this report I have attempted to use a data-driven approach, by extending the Weibull model, to account for sharp changes in the hazard of mortality. Using these extended Weibull models allowed an objective estimation of the possible location of changepoints in the risk of death of HIV-infected patients after they initiate antiretroviral therapy. These parametric models may be superior to semiparametric models (such as the Cox proportional hazards model) in this setting because they make explicit modelling of the underlying mortality risk (baseline hazard). As cited in [4], from a quote attributed to Hjort [12], a "parametric version [of the Cox model] ... if found to be adequate, would lead to more precise estimation of survival probabilities and ... concurrently contribute to a better understanding of the phenomenon under study". Using semiparametric models would have required a much more complex modelling exercise, where factors associated with the changepoints would have to be included among the model predictors.

These analyses show that an early changepoint is likely to exist at about three months after initiation of ART. The presence of this early changepoint is supported by a number of reports. Stringer and colleagues [3], describing the experience of the national antiretroviral therapy programme in Zambia, report that 71% of all deaths in their cohort happened during the first 90 days after initiation of ART. Braitstein and colleagues [1], in a large study of 2,725 HIV-infected persons in 18 antiretroviral programmes in Africa, Asia and South America, report that mortality rates were 14.7% and 10.6% in the first and second month after ART initiation respectively but dropped to 5.1% in months three to six, and then dropped further to 2.7% in months 6–12. These results are similar to our experience summarized in Table 1. The biological plausibility of an initially very high hazard of mortality that rapidly declines over the first few months after initiation of ART is supported by a number of factors. Since all patients involved in this study were treatment-naïve, early drug toxicity may have played a significant role in their ability to adhere to the new medication regimens. In addition, the rapid restoration of immune function immediately after initiation of therapy, may have led to an inflammatory response, what is called an Immune Reconstitution Inflammatory Syndrome (IRIS) that can be fatal for the patient. In a prospective study in South Africa, IRIS occurred in 10% of the patients at a median time 48 days after initiating ART [13], particularly among the most immunosuppressed patients. A number of authors have identified IRIS as having a significant burden in the context of rapid immunological reconstitution in the presence of latent co-infections, particularly in the developing world, where IRIS is "unmasking" a latent existing opportunistic infection or cancer. Given the burden of cryptococcosis deaths in the early period after ART initiation in this study, fatal IRIS-related to inflammatory immune response to this disease may have been present (see Moore et al., 14th CROI presentation http://www.retroconference.org/2007/Abstracts/28827.htm for more information). A relevant case report of fatal cryptococcosis-related IRIS can be found in Seddon and colleagues [14]. The most explicit attribution of early excess death to IRIS is given in Celentano & Beyer [15] who cite a number of investigators discussing fatal cases of IRIS in the context of tuberculosis [16] and cryptococcal antigenemia [17]. The median CD4+ T cell count at ART start (analysis not shown) was 128 cells/ml for this cohort with 25% of the patients having CD4 counts half of that level, implying significant immunosuppression. It has long been recognized that CD4 counts below 200 cells/ml expose HIV-infected patients to a very high risk for opportunistic infections and death, the main reason why therapy is started when CD4 count drops below that level. Given that, on average, patients gain about 100 cells/ml in the first six weeks of treatment and a further 60 cells/ml during the subsequent months of the first year of antiviral therapy [3,18], it is likely that the majority of subjects in the present study reached CD4 counts above 200 cells/ml only after the first three months of starting ART. Consequently, co-infections and morbidities present at the start of ART or acquired in the first months of therapy likely continued to present a significant mortality risk during this period.

We also showed that these generalized Weibull models with changepoints can easily accommodate covariates. In the example provided, men experienced considerably higher mortality compared to women as implied by the 50% higher hazard of death. This has been consistently reported in both the developed and developing world setting [18,19]. In our context, men tend to be more immunosuppressed than women when starting ART. This is because of a number of issues that are beyond the scope of this report. Men also exhibit higher levels of loss to follow-up compared to women [18]. In our cohort, men had lower median CD4 count at ART start than women (analysis not shown).

Despite higher mortality rates among HIV-infected men, both men and women experience high mortality within the first few months after starting ART. This observation in turn implies that, along with gender, patient follow-up and outreach efforts should be directed towards patients that have recently been started on ART: see [20] for description of such a tiered patient outreach protocol.

The existence of a period of moderate mortality risk even past the three-month point, as suggested by the second changepoint, is not surprising given the deep immunosuppression of this study cohort. Nevertheless, persistence of risk up to the first year after starting ART has less clear precedent in the literature, although the mortality rates quoted in some of the aforementioned references suggest that mortality rates stabilize only after about one year from initiation of ART. Evidence from our models is equivocal on this issue. The AIC and BIC criteria applied to the Weibull models with changepoints did favour the model with two changepoints, but their values were close and, as mentioned in Royston and Parmar [4], in the context of a similar class of generalized Weibull models, they should not be used mechanically in selecting the best model. Thus, the evidence for a second changepoint of mortality risk remains weak at present. Additional analyses of similar data with longer follow-up are warranted to elucidate this issue.

Extensions of the Weibull model have been considered by a number of authors. Royston and Parmar [4] have presented a rich class of models that use cubic splines to approximate *s*(*x*; *γ*) in (1) by adding higher-order polynomial terms and one or more "knots" that add flexibility to the shape of the survival curve not available in the simple Weibull model. The methodology has been implemented in [21] and [22] in the STATA software (that also includes similar extensions to the log-logistic survival model). Analysis of the data in this paper (not shown) using the spline models produced virtually identical survival estimates as those generated by the Weibull models with changepoints. A significant advantage of the generalized class of Weibull models of Royston and Parmar is that the resulting hazard plot is continuous unlike the hazard curves produced by the models considered in this work, which have discontinuities at the changepoints. However, the number and placement of the spline knots does not have the same direct biological interpretation as the number and location of changepoints. Thus, the generalized models with splines are less suitable in an effort to estimate the number and location of possible abrupt changes in patient survival, which was the primary goal of this research.

## Conclusion

The hazard of mortality is very high after ART initiation for up to three months, and may persist up to a year after start of treatment. This has strong implications for patient management and may be helpful in refining patient care protocols in this setting by intensifying follow-up of newly treated patients during this period and possibly extending the duration of intensified follow-up for up to one year after start of therapy. Further investigation and re-analysis of data from a number of ongoing studies will be important to authoritatively address this question. The flexibility afforded by these Weibull models will be useful in this endeavor.

## Authors' contributions

The author conceptualized and performed all analyses, interpreted the results and wrote the paper.

## Author's information

CTY is a biostatistician who has been extensively involved with clinical and epidemiological HIV research for 15 years. This has been through the Harvard School of Public Health Statistics and Data Analysis Center of the AIDS Clinical Trials Group, and, more recently, as Director of the East Africa Regional International Epidemiologic Databases to Evaluate AIDS (IeDEA) Consortium at the Indiana University School of Medicine. His research interests in East Africa focus in the use of clinical and research databases to provide a locally relevant evidence basis for medical decision making, health care policy and clinical management of HIV-infected patients cared for and treated in programmes in the region.
